# Severe Thrombocytopenia Caused by Vancomycin in the Intensive Care Unit: A Case Report

**DOI:** 10.3389/fmed.2022.829267

**Published:** 2022-06-09

**Authors:** Xiao-xiao Li, Guan-ru Wang, Chao Li, Na He, Peng Yao, Yin-chu Cheng, Chu-hui Wang, Qing-gang Ge, Min Yi, Zong-yu Wang, Lu-wen Shi, Rong-sheng Zhao

**Affiliations:** ^1^Department of Pharmacy and Department of Intensive Care Unit, Peking University Third Hospital, Beijing, China; ^2^Department of Pharmacy Administration and Clinical Pharmacy, School of Pharmaceutical Sciences, Peking University, Beijing, China

**Keywords:** vancomycin, thrombocytopenia, adverse drug reaction, critically illness, case report

## Abstract

Thrombocytopenia can cause substantial morbidity and mortality in critically ill patients. There are multiple etiology factors and various mechanisms associated with thrombocytopenia, of which drug-induced thrombocytopenia (DITP) deserves attention. Herein, we describe a case of severe thrombocytopenia during intensive care unit (ICU) hospitalization that was likely to be associated with vancomycin. By revealing the process of identifying this case of DITP and reviewing relevant clinical studies, a risk alert of vancomycin-related severe hematotoxicity should be considered.

## Introduction

Thrombocytopenia is defined as a platelet count below the normal lower limit (< 150 × 10^9^/L for adults). Degrees of thrombocytopenia can be further subdivided into mild (100 × 10^9^/L to 150 × 10^9^/L), moderate (5 × 10^9^/L to 99 × 10^9^/L), and severe (< 5 × 10^9^/L). In the setting of immune thrombocytopenia, a platelet count < 3 × 10^9^/L is considered to represent severe thrombocytopenia (1). Thrombocytopenia can cause substantial morbidity and mortality in critically ill patients. The incidence of thrombocytopenia in adult critically ill patients can reach 8.3% to 67.6% (2). Meanwhile, thrombocytopenia is associated with significantly increased bleeding and blood transfusion and even mortality events (3, 4).

There are multiple etiology factors and various mechanisms that can cause thrombocytopenia, and clinical decision-making is quite complicated. Common causes of thrombocytopenia are bone marrow suppression, primary hematological diseases, autoimmune diseases, severe infections, and medications. As an increasing cause of isolated thrombocytopenia, drug-induced thrombocytopenia (DITP) can be attributed to foods and herbal remedies besides medications (5). Among intensive care unit (ICU) patients with new-onset thrombocytopenia, medications can account for 16% (6). DITP is an important clinical problem for physicians, of which drug-dependent antibody mediated platelet destruction is one of the main mechanisms. The most implicated medications include quinine, sulfamethoxazole, penicillin, and linezolid (7). Vancomycin is used for Gram-positive cocci infections, especially in methicillin-resistant *staphylococcus aureus* (MRSA) infection (8). Severe renal impairment, hypersensitivity reactions, and infusion-related reactions caused by vancomycin have been widely recognized in clinical practice, while adverse reactions, such as agranulocytosis and thrombocytopenia, are rare and can be overlooked.

Herein, we present a case with probable vancomycin-induced severe thrombocytopenia during ICU hospitalization. We aim to provide evidence-based references for the diagnosis and treatment of vancomycin-induced thrombocytopenia in critically ill patients.

## Case Presentation

A 64-year-old male patient was admitted to hospital on 21 April 2021 (Day 1, hereafter referred to as D1) with 3-day paroxysmal abdominal cramps, accompanied by diarrhea, nausea, and vomiting. The diagnosis was transverse colon perforation on admission, with rectal mass, septic shock, and hypokalemia. An emergency surgery was performed to repair the laparotomy perforation as well as radical treatment of rectal cancer. After the operation, he was transferred to an intensive care unit (ICU) (D1). The scores of the sequential organ failure assessment (9), acute physiology and chronic health evaluation (10), and Richmond agitation-sedation scale (11) were 8, 19, and −2 scores, respectively. To treat sepsis, ICU physicians implemented bundle strategies including antimicrobial therapy (imipenem and cilastatin sodium injections, 0.5 g q6h), fluid resuscitation (20% human albumin, 40 g; crystoloid solution, 3,000 ml), and sedation therapy (remifentanil, propofol, and midazolam).

**Figure 1:**
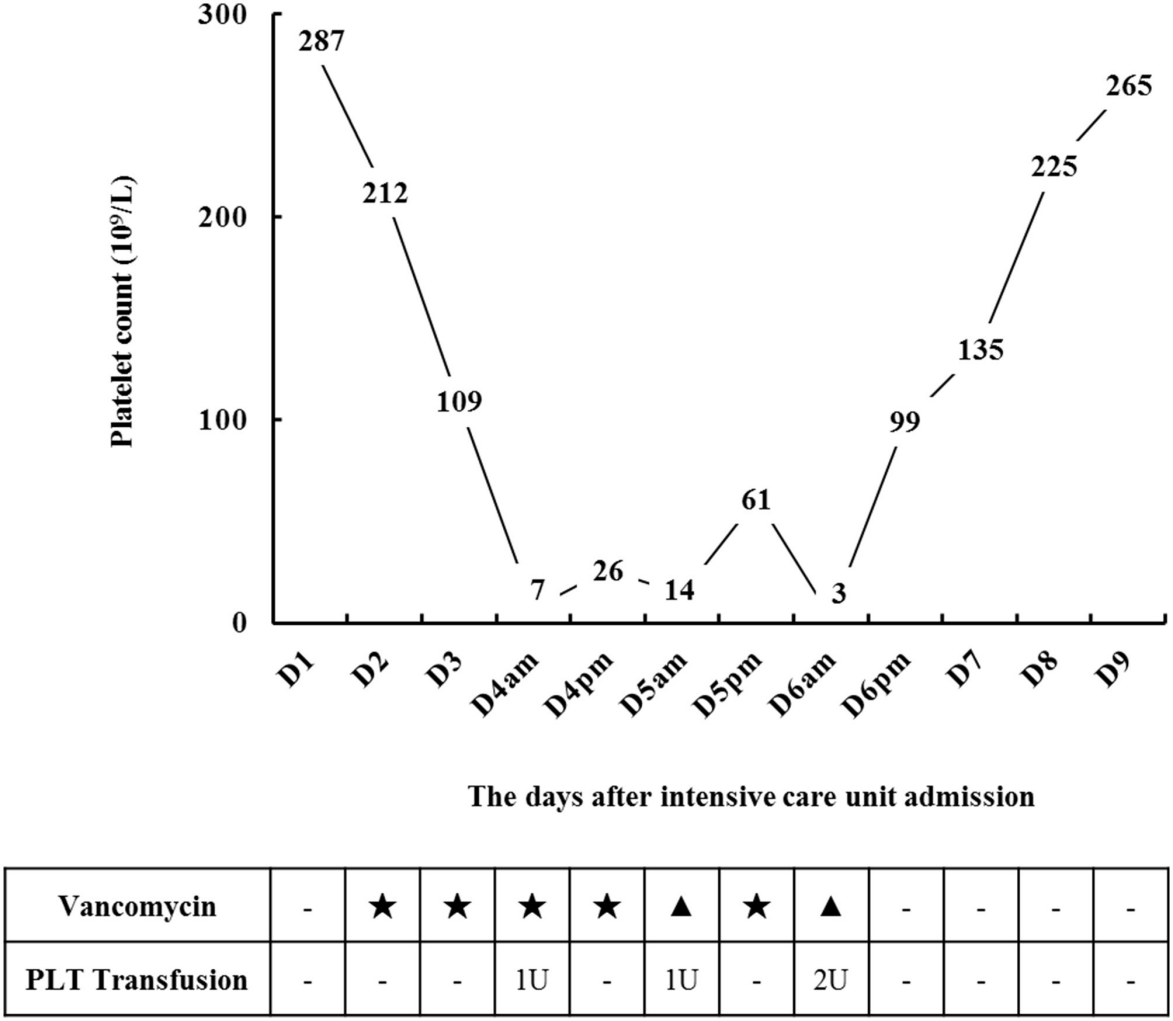
Changes in platelets during treatment. ⋆ means vancomycin exposure, ▴ means discontinuation of vancomycin. PLT, platelet; U, unit, 1U = 2.5 × 10^11^ platelets.

During the ICU hospitalization (D1–D9), his blood routine examinations, body temperature, coagulation, procalcitonin, renal, and liver function laboratory markers were measured, as shown in Table 1. On D2, an operative recording showed a serious fecal contamination in his abdominal cavity. According to the 2019 epidemiological data of the surgical system in our hospital (among 23 samples of Gram-positive cocci isolated from blood bacterial cultures, there were 5 samples of *Enterococcus faecium* and 1 sample of *Enterococcus faecalis*), we considered the possible risk of bloodstream infection of *Enterococcus faecium* or *Enterococcus faecalis*; thus, vancomycin (1 g, q12 h) was administrated intravenously. Here, we used a VCM-TDM tool (Pharmvan, http://www.pharmado.net/) to predict the appropriate vancomycin dosage with a minimal concentration (Cmin) of 10–20 μg/ml (8); and follow-up monitoring of Cmin also proved its feasibility (D4, 11 ug/ml). Unexpectedly, his platelet count (7 × 10^9^/L) severely decreased on D4 (Figure 1).

**Table 1:** Body temperature, blood routine examinations, and procalcitonin (D1–D9)^†^.

**Date**	**Tmax^‡^ (**°**C)**	**Red blood count (10^**12**^/L)**	**White blood count (10^**9**^/L)**	**Platelet count (10^**9**^/L)**	**Neutrophil (10^**9**^/L)**	**Procalcitonin (ng/ml)**	**PT (s)**	**APTT (s)**	**FDP (μg/ml)**	**D-Dimer (μg/ml)**	**Fib (g/L)**	**ALT (U/L)**	**TBIL (μmol/L)**	**DBIL (μmol/L)**	**SCr (μmol/L)**	**Ret (%)**	**Protamine anticoagulant test (3*P* test)**
D1	38.3	4.82	3.99	287	3.09	98.3	15.4	34.8	7.2	1.04	2.21	90	12.7		117		
D2	38.8	3.4	12.08	212	11.41	69.63	19.4	36.8	26.8	4.42	4.79	141	19.2		111		
D3	38.0	3.17	2.28	109	1.95	43.67	15.1	34.2	40.1	6.07	6.15	104	20.4		116		
D4	37.8	2.91	10.21	7 → 26^§^	9.55	17.64	13.1	31.6	28.6	4.50	5.81	74	22.6		78		
D5	38.7	3.44	6.89	14 → 61^§^	5.58	10.87	14.3	29.0	46.3	6.63	4.61	50	21		93		
D6	37.4	3.64	7.49	3 → 99^§^	6.12	4.81	14.9	29.6	48.1	7.04	4.61	48	43.6		69	0.45	negative
D7	37.8	3.32	8.81	135	6.96	2.92	14.2	30.6	53.6	7.21	4.18	71	44.9	28.7	72		
D8	37.9	3.32	9.30	225	7.78	1.76	15.0	27.6	61.7	8.36	4.82	25	45.7	25.8	74		
D9	37.1	3.57	11.96	265	10.75	/	12.4	28.7	52.9	6.67	4.49	36	37.3	21.9	70		

† *Blood sampling was generally undertaken between 4:00-6:00 a.m.; the platelets count was retested within 30 min of finishing the platelet transfusion*.

‡ *Maximum body temperature of the day*.

§ *“ → ” represents changes after platelet infusion*.

*APTT, activated partial thromboplastin time; DBIL, direct bilirubin; FDP, fibrinogen degradation products; Fib, fibrinogen; PT, prothrombin time; Ret, reticulocyte; SCr, serum creatinine*.

**Figure 2:**
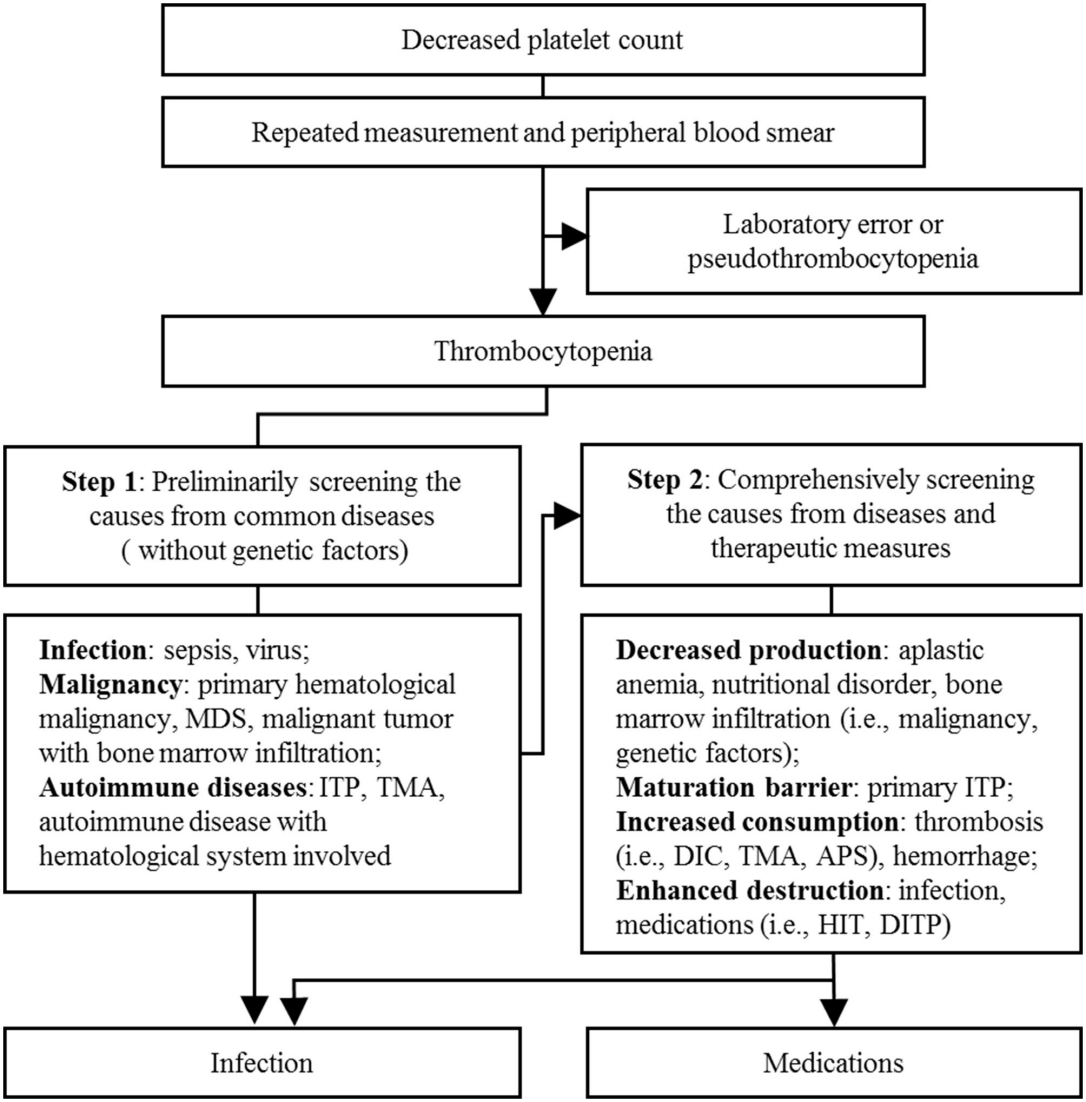
Diagnostic reasoning in thrombocytopenia. APS, antiphospholipid syndrome; DIC, disseminated intravascular coagulation; DITP, drug-induced thrombocytopenia; ITP, immune thrombocytopenia; HIT, heparin-induced thrombocytopenia; MDS, myelodysplastic syndrome; TMA, thrombotic microangiopathy.

To find the causes of thrombocytopenia, as Figure 2 shows, firstly, we excluded the possibility of laboratory error or pseudothrombocytopenia by examining the peripheral blood smear for platelet clumping and repeating the platelet count test. Then, we roughly excluded hematological disease and autoimmune diseases, according to the blood routine test (Table 1) combined with medical history and laboratory markers. For instance, myelodysplastic syndrome or nutritional disorder rarely presents as isolated thrombocytopenia; usually, it combines with the abnormal level in laboratory markers, such as serum iron, vitamin B12, folic acid, reticulocyte count, corpuscular volume, and megakaryocytes. However, no exceptions in above tests occurred in this patient. After that, the result of sonography helped us exclude an enlarged spleen, which might be induced by liver cirrhosis or exhibited as a common clinical manifestation in such primary immune diseases as primary immune thrombocytopenia (ITP). Yet, an alcohol naïve status and a negative result of liver ultrasound helped us further exclude chronic liver diseases. Besides, primary ITP usually persisted for months or years and could be detected in the medical history. We further excluded thrombotic microangiopathy (TMA) as there was no indication for acute kidney injury or hemolytic anemia. Although total bilirubin (TBIL) was elevated, as shown in Table 1, it was dominated by direct bilirubin instead of indirect bilirubin, and fragmented red blood cells were not found in the blood smear. Meanwhile, hepatic failure and disseminated intravascular coagulation (DIC) were put aside according to liver function laboratory and coagulation markers. Although there were fluctuations in coagulation markers, the trend was not synchronized with the platelet count trend, and the protamine anticoagulant test (3P test) suggested a low probability of DIC. Thus, thrombocytopenia was a major concern, which might be caused by infection or medications. After transfusion of platelets (1 U) and blood cells (2 U) on D4, his platelet count recovered a little (26 × 10^9^/L). However, it went down again in the morning of D5 (14 × 10^9^/L). Patients with severe infection, such as sepsis, generally have laboratory abnormalities indicative of systemic infection (leukocytosis or neutropenia) and ongoing coagulation (prolonged prothrombin time and activated partial thromboplastin time, low fibrinogen, and elevated D-dimer). In this case, we found that the patient's severe infection was alleviated by the above markers during D1–D5. Therefore, thrombocytopenia was less likely caused by infection, and we started to focus on medications. Among all the medications used between D1–D5, there was no administration records of systemic heparin or linezolid, and antitumor therapy had not been started. Vancomycin aroused suspicion as a previous study had shown that it could cause thrombocytopenia at the initial exposure without prior sensitization (12). Thus, vancomycin was discontinued on D5 in the morning (the overall diagnostic diagram is shown in Figure 2), and a platelet transfusion (1U) was given to prevent hemorrhage.

In the evening of D5, as the body temperature increased to 38.7°C, the patient had to be re-administered vancomycin. As a result, his platelet count reduced from 61 × 10^9^/L to 3 × 10^9^/L within 8 h rapidly. He had to receive another platelet (2 U) transfusion in the morning of D6. As the drainage fluid culture suggested only a extended spectrum β-lactamase-negative (ESBL-) *Escherichia coli* infection, we completely discontinued vancomycin but kept the patient on imipenem and cilastatin sodium. The platelet count returned to 135 × 10^9^/L on D7 and did not drop again after that. He was transferred back to the general ward after being extubated (D9). On D14, he stopped using antibiotics and was discharged with a better health condition. According to the Naranjo adverse reaction evaluation scale (Table 2) (13), the total score was 6, and the relationship between vancomycin and thrombocytopenia was judged as “probable.”

**Table 2:** Naranjo adverse drug reaction probability scale.

**Questions**	**Scores**			**Results**
	**Yes**	**No**	**Not known**	
1. Are there previous conclusive reports on this reaction?	+1	0	0	+1
2. Did an adverse event appear after the suspected drug was given?	+2	−1	0	+2
3. Did the adverse reaction improve when the drug was discontinued or a specific antagonist was given?	+1	0	0	+1
4. Did the adverse reaction appear when the drug was readministered?	+2	−1	0	+2
5. Are there alternative causes that could have caused the reaction?	−1	+2	0	−1
6. Did the reaction reappear when a placebo was given?	−1	+1	0	0
7. Was the drug detected in any body fluid in toxic concentrations?	+1	0	0	0
8. Was the reaction more severe when the dose was increased, or less severe when the dose was decreased?	+1	0	0	0
9. Did the patient have a similar reaction to the same or similar drugs in any previous exposure?	+1	0	0	0
10. Was the adverse event confirmed by any objective evidence?	+1	0	0	+1
Total score				6

## Discussion

DITP diagnosis is complicated, owing to the multiple causes of acquired thrombocytopenia in ICU patients. Among thrombocytopenic patients who present with severe infection, DITP is a very important distinction to make. Firstly, we roughly excluded primary blood diseases and autoimmune diseases through medical history and laboratory examination results. Then, TMA, hepatic failure, and DIC were excluded according to renal and liver function laboratory and coagulation markers. At last, we focused on the infection and medication factors. As a priority factor suspected in thrombocytopenia, the severe infection had been alleviating (Table 1), while the platelet count decreased. Thus, vancomycin caught our attention. It offered further clinical evidence as thrombocytopenia appeared when vancomycin was re-administered and disappeared when vancomycin was discontinued.

Thrombocytopenia occurs in 7.1% of patients treated with vancomycin (14); however, it could be easily overlooked as its description and incidence have not been labeled. There have been some case reports of vancomycin-induced thrombocytopenia before. Back in 1985, Walker presented a 48-year-old female patient with secondary peritonitis after peritoneal dialysis. During the treatment period, vancomycin (500 mg, i.v.gtt.) was given first, followed by intraperitoneal injection of 120 mg/day, and the patient showed significant thrombocytopenia 6 days later (15). Howard reported in 1997 that a 58-year-old male patient admitted to hospital due to osteomyelitis caused by MRSA, manifested thrombocytopenia after vancomycin administration (16). In 2013, a systematic review, including pediatric patients (<18 years old) (17), found that 32 substances had potential pathogenic effects in thrombocytopenia, including vancomycin (in 3 of 21 cases). In 2017, 30 case reports with 30 patients were included in a scoping review that reported vancomycin-induced thrombocytopenia (18). It can occur on the 1st to 10th day after drug exposure. The time window for the platelet count dropping to the lowest point ranged from 4 h to 10 days after drug exposure. The median platelet count often dropped to < 20 × 10^9^/L (17, 19), with those < 2 × 10^6^/L to 1 × 10^8^/L suffering from bleeding (20). The degree and occurrence time (the time for the platelet count to drop to 7 × 10^9^/L was 2 days of vancomycin use) of thrombocytopenia in our case were basically consistent with the previous reports.

Usually, DITP was alleviated after vancomycin withdrawal, with the severe case requiring platelet infusion (18). There is no evidence for the efficacy of immunosuppression in treating DITP. In previous reports, vancomycin was discontinued in 29 of 30 patients, and the platelet count in 17 patients recovered within 5–6 days (12, 19). In this process, patients' infection control needs to be taken into account when discontinuing vancomycin. In our case, luckily, the patient was definitely infected with *Escherichia coli* (ESBL-), and we decisively discontinued vancomycin after thrombocytopenia was observed again. As thrombocytopenia leads to an increased risk of bleeding, the transfusion of platelets is a common management plan, although it does not always result in the expected platelet count increase of affected patients (67% of 20 patients were transfusion-resistant) (18). In this case, platelet infusion was given when the platelet count was at 7 × 10^9^/L and 14 × 10^9^/L, and a platelet 2U infusion was given at 3 × 10^9^/L.

There are three limitations in this study. Firstly, only postoperative drainage fluid was collected for culture specimens after starting antimicrobial therapy, but no blood samples were obtained before at emergency. We should continue to improve the implementation of the sepsis bundle, emphasizing the importance of retaining blood samples to monitor and analyze the bacteria before empirical antibiotic treatment (21). Secondly, we could not confirm whether the cause was DITP or not, as our hospital does not perform tests of drug-dependent platelet reactive antibodies (22). In order to better identify DITP and improve patient prognosis, it is suggested to improve the accessibility of platelet reactive antibody technology in the future. Thirdly, based on a high risk of bleeding caused by the severe thrombocytopenia as well as the willingness of the patient's family members, hematological diseases could not be entirely excluded as neither bone marrow puncture nor bone biopsy for the patient were carried out.

## Conclusions

In this study, we report a patient with probable vancomycin-induced severe thrombocytopenia during ICU hospitalization. By revealing the process of identifying this case of DITP and systematically reviewing relevant clinical studies, a risk alert of vancomycin-related severe hematotoxicity should be considered.

## Data Availability Statement

The raw data supporting the conclusions of this article will be made available by the authors, without undue reservation.

## Ethics Statement

Ethical review and approval was not required for the study on human participants in accordance with the local legislation and institutional requirements. The patients/participants provided their written informed consent to participate in this study. Written informed consent was obtained from the individual(s) for the publication of any potentially identifiable images or data included in this article.

## Author Contributions

X-xL: conceptualization, investigation, data curation, visualization, and writing-original draft. G-rW: data curation, visualization, and writing-original draft. CL: conceptualization, visualization, and writing-review and editing. NH: conceptualization and writing-review and editing. PY and C-hW: writing-review and editing. Y-cC: conceptualization. Q-gG, MY, and Z-yW: investigation. L-wS and R-sZ: supervision and validation. All authors took part in the final version for submission and accept overall accountability for accuracy and integrity of the manuscript.

## Funding

This work was supported by the National Natural Science Foundation of China [Grant No. 71904002, receiver: Y-cC], as well as Peking University Medicine Fund of Fostering Young Scholars' Scientific and Technological Innovation, which was established by Fundamental Research Funds for the Central Universities [Grant No. BMU2022PYB011, receiver: X-xL].

## Conflict of Interest

The authors declare that the research was conducted in the absence of any commercial or financial relationships that could be construed as a potential conflict of interest.

## Publisher's Note

All claims expressed in this article are solely those of the authors and do not necessarily represent those of their affiliated organizations, or those of the publisher, the editors and the reviewers. Any product that may be evaluated in this article, or claim that may be made by its manufacturer, is not guaranteed or endorsed by the publisher.

## Abbreviations

APTT: activated partial thromboplastin time
APS: antiphospholipid syndrome
DBIL: direct bilirubin
DIC: disseminated intravascular coagulation
DITP: drug-induced thrombocytopenia
ESBL-: extended spectrum β-lactamase-negative
ICU: intensive care unit
ITP: immune thrombocytopenia
FDP: fibrinogen degradation products
Fib: fibrinogen
HIT: heparin-induced thrombocytopenia
MDS: myelodysplastic syndrome
MRSA: methicillin-resistant staphylococcus aureus
PLT: platelet count
PT: prothrombin time
Ret: reticulocyte
SCr: serum creatinine
TMA: thrombotic microangiopathy.
